# Red CYTED-RITMOS: hacia la búsqueda de soluciones para fomentar la salud móvil en América Latina

**DOI:** 10.26633/RPSP.2017.33

**Published:** 2017-03-22

**Authors:** Francesc Saigí-Rubió, David Novillo-Ortiz, John D Piette

**Affiliations:** 1 Estudios de Ciencias de la Salud Universitat Oberta de Catalunya Barcelona España Estudios de Ciencias de la Salud, Universitat Oberta de Catalunya, Barcelona, España.; 2 Organización Panamericana de la Salud Oficina de Gestión del Conocimiento, Bioética e Investigación Washington D.C. Estados Unidos de América Organización Panamericana de la Salud, Oficina de Gestión del Conocimiento, Bioética e Investigación, Washington D.C., Estados Unidos de América.; 3 Department of Health Behavior and Health Education School of Public Health, University of Michigan, Ann Arbor Ann ArborMichigan Estados Unidos de América Department of Health Behavior and Health Education, School of Public Health, University of Michigan, Ann Arbor, Michigan, Estados Unidos de América.

**Keywords:** Telemedicina, poblaciones vulnerables, tecnología biomédica, América Latina, Telemedicine, vulnerable populations, biomedical technology, Latin America, Telemedicina, populações vulneráveis, tecnologia biomédica, América Latina

## Abstract

El ámbito de las tecnologías móviles aplicadas a la salud (mSalud) es una tendencia en auge a nivel mundial que ha generado enormes expectativas para paliar los problemas de prestación de servicios médicos y de salud pública ocasionados por la escasez de recursos y el reducido número de especialistas. Las numerosas posibilidades que aportan las tecnologías móviles, junto con su facilidad de uso, han atraído el interés tanto de los gobiernos políticos como el de las universidades. Es el caso de la Red Iberoamericana de Tecnologías Móviles y Salud (Red CYTED-RITMOS). Como resultado del primer año de actividad de la Red, en octubre de 2015 tuvo lugar en Barcelona el Internacional Workshop RITMOS con el objeto de presentar las áreas prioritarias de América Latina donde podrían desarrollarse proyectos de investigación, desarrollo e innovación (I+D+i) en salud móvil y posibles soluciones. El objetivo de este trabajo es mostrar las potencialidades y la aplicabilidad de la mSalud en la región de las Américas.

## Abstract (en)

The area of mobile technologies applied to health (mHealth) is a growing worldwide trend that has generated enormous expectations for the mitigation of problems related to medical services delivery and public health stemming from a lack of resources and the limited number of specialists. The numerous opportunities offered by mobile technologies, together with their ease of use, have attracted the interest both of governments and universities. This is the case of the Ibero-American Mobile Technologies and Health Network (CYTED-RITMOS, Spanish acronym). As a result of the network’s first year of activity, in October 2015 the RITMOS International Workshop was held in Barcelona to present the priority areas in Latin America where research, development, and innovation (R&D+i) projects on mobile health could be carried out and possible solutions found. The objective of this article is to present the potentialities and applicability of mHealth in the Region of the Americas.

## RESUMO

O âmbito das tecnologias móveis aplicadas à saúde (mSaúde) é uma tendência crescente a nível mundial que tem gerado enormes expectativas, com a promessa de mitigar os problemas na prestação de serviços médicos e de saúde pública ocasionados pela escassez de recursos e pelo baixo número de especialistas. O grande número de possibilidades geradas pelas tecnologias móveis, juntamente com sua facilidade de uso, têm atraído o interesse tanto de governos como de universidades. É o caso da Rede Ibero-Americana de Tecnologias Móveis e Saúde (Rede CYTED-RITMOS). Como resultado do primeiro ano de atividade da Rede, em outubro de 2015 foi realizado em Barcelona o Seminário Internacional RITMOS, com o objetivo de apresentar as áreas prioritárias da América Latina onde poderiam ser desenvolvidos projetos de pesquisa, desenvolvimento e inovação (P&D+i) em saúde móvel e possíveis soluções. O objetivo deste trabalho é mostrar os potenciais e a aplicabilidade da mSaúde na Região das Américas.

El ámbito de las tecnologías móviles aplicadas a la salud —denominadas mSalud— es una tendencia en auge a nivel mundial que ha generado enormes expectativas para paliar los problemas de prestación de servicios médicos y de salud pública ocasionados por la escasez de recursos y el reducido número de especialistas, tanto en países desarrollados como en países emergentes (1). Por un lado, estas tecnologías podrían convertirse en un gran aliado de los profesionales a la hora de llevar a cabo su actividad asistencial. Por el otro, podrían facilitar que la población participara de forma activa y responsable en el cuidado de su salud y prevención de enfermedades (2).

No obstante, la evolución de la mSalud debe ir en consonancia con las necesidades y de los recursos de los que disponga cada región (3). Es por ello que, para garantizar el éxito en la implementación de un servicio de mSalud, es básico determinar las prioridades y necesidades del servicio enmarcadas en el contexto de la realidad sociocultural, sociosanitaria y de recursos del ámbito (4). La realización de un análisis holístico, entendido aquí como análisis de la realidad de un modo global o integral, es esencial para comprender mejor las necesidades, las condiciones y los recursos más relevantes para el proceso de integración del servicio de mSalud en el correspondiente escenario (infraestructura, financiación, recursos humanos y organizativos, estándares y cuestiones legales, éticos y privacidad de la información).

Las numerosas posibilidades que aportan las tecnologías móviles aplicadas a la salud, junto con su facilidad de uso han atraído el interés tanto de los gobiernos políticos como de las universidades. Es el caso de la Red Iberoamericana de Tecnología Móviles y Salud (Red CYTED-RITMOS - 515RT0498), una red internacional liderada por la Universitat Oberta de Catalunya (UOC) y compuesta por 17 grupos de investigación de seis países (Argentina, Bolivia, Chile, Colombia, Ecuador y España), la Organización Panamericana de la Salud/Organización Mundial de la Salud (OPS/OMS), Médicos Sin Fronteras (MSF), Telefónica, la Fundación Mobile World Capital Barcelona (FMWCB) y la Universidad de Michigan (UM), que pretende fomentar la investigación y el desarrollo de la mSalud en América Latina (AL). Como resultado del primer año de actividad de la Red, en octubre de 2015 tuvo lugar en Barcelona el Internacional Workshop RITMOS con el objeto de presentar las áreas prioritarias de AL en las cuales podrían desarrollarse proyectos de investigación, desarrollo e innovación (I+D+i) en salud móvil y soluciones ya implantadas con resultados satisfactorios. El objetivo de este trabajo es mostrar las potencialidades y la aplicabilidad de la mSalud en AL.

## RESULTADOS

### Apoyo a los pacientes con enfermedades crónicas a través de la mSalud en América Latina

Hasta la fecha, hay poca evidencia científica sobre los beneficios de las aplicaciones para teléfonos inteligentes (*apps*) (5), y todavía, muchas personas en AL no cuentan con un teléfono móvil inteligente (*smartphone*). Por el contrario, las intervenciones con base en mensajes de texto (*short message service* o SMS) han demostrado ser una opción muy útil cuando se trata de apoyar a los pacientes a dejar de fumar (6), para controlar los factores de riesgo en pacientes con enfermedades cardiovasculares (7), para controlar la adherencia al tratamiento antiviral en pacientes con virus de inmunodeficiencia humana (8) e incluso para el tratamiento de enfermedades como la diabetes (9) o la tuberculosis (10).

También es el caso de la respuesta de voz interactiva (IVR por sus siglas en inglés), a través de la cual han desarrollado el Programa *CarePartner* en Estados Unidos, un programa que pretende cambiar el comportamiento de los pacientes para mejorar su estado de salud. Gracias a una serie de contenidos desarrollados por expertos, se realizan una serie de llamadas automatizadas a través de las cuales los pacientes marcan respuestas a las preguntas recibidas por voz y reportan información sobre su salud. A cambio, reciben información puntual sobre la evolución de su autocuidado. A su vez, el centro asistencial recibe una alerta acerca de síntomas y posibles signos preocupantes, con las posibilidades que esto conlleva a la hora de anticiparse al posible episodio agudo. Además, el programa cuenta con la participación de familiares del paciente, los cuales reciben información actualizada sobre qué pueden hacer para ayudar a su ser querido. Es precisamente esta implicación de los familiares lo que determina incrementos importantes en los resultados de salud de los pacientes más vulnerables y en los contextos más remotos, y que este canal de afecto puede ser de gran utilidad en países de AL, donde la familia desempeña un rol importante en la vida de una persona con una enfermedad crónica (11).

Este programa se ha desarrollado para el tratamiento de pacientes con enfermedades como la diabetes, la hipertensión, la depresión, así como pacientes con problemas cardíacos, con cáncer suprarrenal, en quimioterapia, posthospitalizados, con problemas hepáticos y derrame cerebral y ya ha sido evaluado en cuatro países de AL (Honduras (12), Bolivia (13), México y, más recientemente, en Colombia) además de Estados Unidos de América. En un estudio sobre la factibilidad de la tecnología móvil con pacientes diabéticos en Honduras (14) se halló que a la inmensa mayoría de los pacientes (83%) les fue fácil responder a las llamadas automatizadas, que el servicio les proporcionó información útil (86%), y que el servicio les ayudaba “mucho” (70%). En términos globales, el servicio permitía mejorar el apoyo al autocuidado y una disminución significativa de los niveles de glicemia. Además, en un ensayo aleatorizado realizado en Honduras y México, se mostró que un servicio semejante puede ayudar a los pacientes hipertensos a controlar su tensión sanguínea y mejorar otros resultados de salud (11).

En aras de encontrar los factores de riesgo que limiten el uso de esta tecnología por parte de los pacientes, se lanzó una encuesta a 1 000 usuarios de la Paz, Bolivia (15), preguntándoles sobre el uso de la telefonía móvil. Noventa y seis por ciento de la población comprendida entre las edades 18–29 años tenía un celular y 32% tenía un *smartphone*. Por otro lado, 82% de los indígenas tenía un celular, mientras que 72% de los analfabetos tenía uno. En relación a la población comprendida en la tercera edad, solo 7% de esta población disponía de un teléfono inteligente, mientras que el porcentaje aumentaba a 31% para la población igual o mayor de 50 años. A pesar de la diferencia económica del país, se encontró que 74% de la población comprendida entre las edades de 30–49 años usaban mensajes de texto.

### Áreas de mejora en América Latina para desarrollar proyectos de investigación, desarrollo e innovación en mSalud

Ante la necesidad de impulsar el lineamiento de la mSalud en AL, es necesario saber en primer lugar cuáles son las prioridades de los países de la región de las Américas. Conocer cuáles son las diez causas de muerte más frecuente sería la primera, mientras que los Objetivos de Desarrollo Sostenible aprobados en setiembre de 2015 constituyen la siguiente prioridad.

En una encuesta desarrollada por la OMS, se preguntó a los gobiernos de ocho países de la región de las Américas cuáles eran las barreras que ellos habían detectado a la hora de abordar el campo de la mSalud y la eSalud (definida aquí como una práctica emergente en la intersección de la informática médica, la salud pública y las empresas) (16). La falta de personal calificado fue la primera barrera detectada, superando al de las infraestructuras, que ocupaba el segundo lugar. Además, la falta de un modelo de negocio y la falta de compromiso político junto con la falta de sostenibilidad económica fueron otras barreras identificadas que obstaculizan la difusión de sendos servicios (17). De allí la necesidad de convencer a los gobiernos de apostar por proyectos de estado de largo plazo, a pesar de sus cortas vidas a nivel político. A su vez, se determinó la necesidad de establecer nuevas políticas de formación a la hora de implantar soluciones mSalud, junto con una gestión del cambio eficiente con la implicación de los órganos políticos. Las políticas de formación deben ir dirigidas tanto a los profesionales sanitarios como a los ciudadanos. A los profesionales, en la adquisición de nuevas habilidades y conocimientos vinculados con su actividad asistencial haciendo uso de estas tecnologías inalámbricas, y muy especialmente cuando se encuentran en zonas rurales y alejadas de las grandes ciudades o núcleos del conocimiento. A los ciudadanos, para paliar el factor de riego que supone la falta de educación en salud de la población, tanto para la prevención como la gestión de las enfermedades.En la región de las Américas, la penetración de acceso a internet se sitúa alrededor de 66% (18). Sin embargo, su uso en el campo de la salud es todavía reducido, muy a pesar de la alta penetración de la telefonía móvil, que se sitúa en 108% (18). La hipótesis aquí es que esta educación en salud debería ir asociada de un cambio de comportamiento del ciudadano y facilitar acciones como el uso correcto de los servicios de salud o mejorar la adherencia a posibles tratamientos.

En una encuesta del 2011 dirigida a los países de la región de las Américas se detectó que los proyectos de mSalud estaban enfocados principalmente a situaciones de emergencias médicas y desastres (19). En relación a las barreras, se detectó una falta de definición clara de las prioridades y que los proyectos tampoco tenían su correlación con la legislación del país. La barrera legal impide que haya mayor cooperación técnica entre países a la hora de intercambiar datos de salud. Por otra parte, existe la necesidad de modelos de gobernanza y con una sostenibilidad de 10 a 15 años vista. Es por esto que en la encuesta de 2015 de la OPS/OMS se preguntara por las políticas en las que basan los proyectos de mSalud, así como por las estrategias y por la regulación (20). Precisamente la falta de regulación es otra de las realidades puestas de manifiesto en la región de las Américas.

### Posibles aplicaciones para fomentar la mSalud en América Latina

Durante las jornadas del International Workshop RITMOS 2015 en Barcelona se presentaron varias aplicaciones móviles y servicios. Es el caso de la gestión remota de pacientes crónicos por la empresa Telefónica, ya en funcionamiento, o la plataforma de análisis intervencional terapéutico para la evaluación y predicción farmacológico en hipertensión arterial de la Universidad de Manizales (Colombia). Otro servicio prometedor presentado por la Universidad Nacional de Rosario (Argentina) fue la estación móvil (TES, *Transportable Examination Station*) compuesto por los dispositivos necesarios para la realización de chequeos médicos y consultas con especialistas ubicados en los centros hospitalarios. El TES permite la atención asistencial en situaciones y escenarios de emergencias médicas.

Otras soluciones presentadas fueron las desarrolladas por la Universidad de Antioquia (Colombia) para brindar apoyo médico y emocional a los afectados de minas antipersonas y munición abandonada sin explotar; el sistema de información de salud de Médicos Sin Fronteras (MSF), que facilita la entrada, validación, análisis y transmisión de datos a lo largo de todos los niveles de la organización en los contextos más remotos; unas aplicaciones de la Universidad de Antioquia para concienciar y prevenir las enfermedades por vectores como el dengue o la malaria en zonas aisladas en países en vías de desarrollo; y un sistema para potenciar el aprendizaje de destrezas motoras en cirugías de invasión mínima utilizando un entorno virtual en línea para practicar en cualquier lugar y momento (proyecto liderado por la UOC y llevado a cabo en la Universidad de Caldas (Colombia) (21).

## CONCLUSIONES

Los últimos años han sido cruciales en los avances realizados en el ámbito de las tecnologías móviles que, aplicadas a la salud, son consideradas como un recurso potencial para mejorar la calidad y el acceso a la asistencia sanitaria. Según las evidencias presentadas en el Internacional Workshop RITMOS 2015, podrían utilizarse herramientas mSalud para mejorar la calidad del diagnóstico, tratamiento y monitorización, así como el apoyo del autocuidado y mejorar la calidad y la esperanza de vida de la población más necesitada en América Latina.

Sin embargo, y a pesar de la creciente oferta de aplicaciones, todavía están lejos de suponer un sistema integrado en la actual asistencia sanitaria. Aún falta mucha evidencia científica que corrobore los resultados obtenidos. A su vez, es necesario incentivar a los órganos políticos para que se impliquen en el establecimiento de un plan de incentivación a favor de la mSalud y dirigido a todos los actores del sistema, con la asignación de un papel destacado a los pacientes.

### Financiamiento.

John Piette recibe apoyo del grant #P30DK092926 del National Institute of Diabetes and Digestive, and Kidney Disease.

### Declaración.

Las opiniones expresadas en este manuscrito son responsabilidad del autor y no reflejan necesariamente los criterios ni la política de la *RPSP/PAJPH* y/o de la OPS.
